# Standardized image interpretation and post-processing in cardiovascular magnetic resonance - 2020 update: Society for Cardiovascular Magnetic Resonance (SCMR): Board of Trustees Task Force on Standardized Post-Processing

**DOI:** 10.1186/s12968-020-00610-6

**Published:** 2020-03-12

**Authors:** Jeanette Schulz-Menger, David A. Bluemke, Jens Bremerich, Scott D. Flamm, Mark A. Fogel, Matthias G. Friedrich, Raymond J. Kim, Florian von Knobelsdorff-Brenkenhoff, Christopher M. Kramer, Dudley J. Pennell, Sven Plein, Eike Nagel

**Affiliations:** 1 Department of Cardiology and Nephrology, Working Group on Cardiovascular Magnetic Resonance, Experimental and Clinical Research Center, a joint cooperation between the Charité Medical Faculty and the Max-Delbrueck Center for Molecular Medicine, and HELIOS Klinikum Berlin Buch, Schwanebecker Chaussee 50, 13125 Berlin, Germany; 2 University of Wisconsin School of Medicine and Public Health, Madison, USA; 3 Department of Radiology of the University Hospital Basel, Basel, Switzerland; 4 Imaging, and Heart and Vascular Institutes, Cleveland Clinic, Cleveland, OH USA; 5 Department of Radiology, Children’s Hospital of Philadelphia, University of Pennsylvania School of Medicine, Philadelphia, PA USA; 6 Departments of Medicine and Diagnostic Radiology, McGill University, Montreal, QC Canada; 7 Duke Cardiovascular Magnetic Resonance Center, and Departments of Medicine and Radiology, Duke University Medical Center, Durham, NC USA; 8 Department of Cardiology, Academic Teaching Hospital Agatharied of the Ludwig-Maximilians-University Munich, Hausham, Germany; 9 Departments of Medicine and Radiology and the Cardiovascular Imaging Center, University of Virginia Health System, Charlottesville, VA USA; 10 Royal Brompton Hospital, and Imperial College, London, UK; 11 Leeds Institute for Genetics Health and Therapeutics & Leeds Multidisciplinary Cardiovascular Research Centre, University of Leeds, Leeds, UK; 12 Institute for Experimental and Translational Cardiovascular Imaging, DZHK (German Centre for Cardiovascular Research) Centre for Cardiovascular Imaging, partner site RheinMain, University Hospital Frankfurt, Frankfurt am Main, Germany

**Keywords:** Magnetic resonance imaging, Heart, Recommendations, Image interpretation, Post-processing

## Abstract

With mounting data on its accuracy and prognostic value, cardiovascular magnetic resonance (CMR) is becoming an increasingly important diagnostic tool with growing utility in clinical routine. Given its versatility and wide range of quantitative parameters, however, agreement on specific standards for the interpretation and post-processing of CMR studies is required to ensure consistent quality and reproducibility of CMR reports. This document addresses this need by providing consensus recommendations developed by the Task Force for Post-Processing of the Society for Cardiovascular Magnetic Resonance (SCMR). The aim of the Task Force is to recommend requirements and standards for image interpretation and post-processing enabling qualitative and quantitative evaluation of CMR images. Furthermore, pitfalls of CMR image analysis are discussed where appropriate. It is an update of the original recommendations published 2013.

## Preamble

Cardiovascular magnetic resonance (CMR) has evolved into a gold standard non-invasive imaging tool in cardiovascular medicine, especially for visualizing and quantifying cardiovascular anatomy, volumes, and function, as well as for myocardial tissue characterization. CMR has unique capabilities in the diagnostic workup of suspected cardiovascular disease. It continues to expand its utility in day-to-day clinical practice. Given its versatility and wide range of quantitative parameters, agreement on specific standards for the image interpretation and post-processing of CMR studies is required to ensure consistent quality and reproducibility of CMR reports. This document addresses this need by updating the 2013 consensus recommendations developed by the Task Force for Post-Processing of the Society for Cardiovascular Magnetic Resonance (SCMR) [1]. The aim of the document is to recommend requirements and standards for image interpretation and post-processing, enabling qualitative and quantitative evaluation of CMR images. Furthermore, pitfalls of CMR image analysis are discussed where appropriate. The Task Force is aware that for some of the recommendations the body of evidence is limited. Thus, this document represents an expert consensus providing guidance based on the best available evidence at present as endorsed by the SCMR. As CMR continues to develop, updated recommendations for image acquisition, interpretation and post-processing will be provided by online appendices when needed and updated Task Force papers.

The recommendations are considered for the application of CMR in clinical routine in adult patients. For some applications, quantification is considered as providing added information but is not mandatory (e.g., perfusion), whereas for others quantification is required for all clinical reports (e.g., T2* assessment in iron overload). In general, the intention of this Task Force is to describe the scenarios in which quantitative analysis should be performed and how it is performed. Quantification itself is a moving target as artificial intelligence approaches to quantification are presently being instituted within CMR analysis software programs and will impact techniques in this arena in the future. The recommendations respect societal recommendations for structured reporting of cardiovascular imaging studies in general (ACCF / ACR / AHA / ASE / ASNC / HRS / NASCI / RSNA / SAIP / SCAI / SCCT / SCMR) [2] and specifically for CMR studies (SCMR) [3]. The recommendations do not supersede clinical judgment regarding the contents of individual interpretation of imaging studies. The Task Force made every effort to avoid conflicts of interest and, where present, to disclose potential conflicts.

## General recommendations

The recommendations listed in this section apply to the acquisition and post-processing of all CMR data. CMR studies should be performed for recommended indications. Data acquisition and reporting should conform to the recommendations of SCMR [3, 4]. Consistent methods of acquisition and measurements are essential for serial evaluation of changes over time. Standardized structured reports with tables of measurements are helpful for reporting follow-up examinations. Any analysis should be performed using uncompressed or lossless compressed Digital Imaging and Communications in Medicine (DICOM) source images. Factors like type of sequence, spatial resolution, contrast agent and kinetics may influence visual and quantitative analysis and should be considered. Quantitative values should only be provided based on adequate image quality. Since there are no objective criteria for inadequate images, this determination needs to rely on the experience of the reporting physician. Readers should have adequate training and clinical experience that includes normal datasets to avoid over-interpretation of normal variants. The identity and responsibility of the reader should be appropriately documented in the report. Furthermore, the reader of clinical data is also responsible for the use of adequate post-processing hardware and software. The general requirements include:

1. Workstation and screen of adequate specification and resolution (as per the specifications of the post-processing software)
2. Post-processing software with regulatory approval for use in patients, ideally providing the following tools:
  - Full DICOM send/retrieve functionality, network connection with local Picture Archiving and Communication System (PACS) or server solution with compliant patient security properties
  - View all short-axis cines as movies in a single display, zoom, pan and change contrast for single images as well as image series
  - Perform endocardial and epicardial contour tracings on cines
  - Correct for atrioventricular annular location from the long-axis slice onto the most basal left ventricular (LV) short-axis location in contour tracings
  - Cross-referencing of structures for confirmation of slice position and anatomy
  - Simultaneously view cine, late gadolinium enhancement (LGE) and/or perfusion images from the same location
  - Simultaneously view short- and long-axis images of the same region
  - Simultaneously view images of the approximate same location on the current and prior study for serial studies
  - Perform quantitative signal intensity (SI) and derived analyses
  - Perform standardized segmentation of the myocardium according to the segment model of the American Heart Association (AHA) [5]
  - Measure flow velocities and flow volumes
  - Manually correct or enter heart rate, blood pressure, height, weight, body surface area
  - Calculate volumes in stacked or 3D datasets with minimal user interaction, including and excluding trabecular tissue and papillary muscles from the LV volume [6]
  - Document important findings in screenshots for the report
  - For evaluation of angiography the software ideally provides the following tools:
    i) 3D multiplanar and maximum intensity projection (MIP) capabilities
    ii) Volume rendering and surface shaded reconstructions optional for reporting but not mandatory for quantitative analysis
    iii) Measurement of distances and areas in 3D-MR angiography (MRA) images
    iv) MIP reconstruction based on non-subtracted or subtracted 3D-MRA datasets
    v) Multiplanar reformatting (MPR)

## Left ventricular chamber assessment

### Visual analysis

- Before analyzing the details, review all cines in cine mode, validate observations from one plane with the others, and check for artifacts, especially in patients with irregular heart rates.
- Dynamic evaluation of global LV function: Interpretation of both ventricular chambers, in concert with extracardiac structures including assessment for hemodynamic interaction between the two chambers (e.g., shunts, evidence of constrictive physiology).
- Assessment of LV function from a global and segmental perspective. Segmental wall motion is based on segmental wall thickening during systole. Wall motion is categorized as: hyperkinetic, normokinetic, hypokinetic, akinetic, dyskinetic.
- In presence of segmental wall motion abnormalities, use of standard LV segmentation nomenclature corresponding to the supplying coronary artery territories is recommended [3, 5, 7].

### Quantitative analysis

**Fig. 1:**
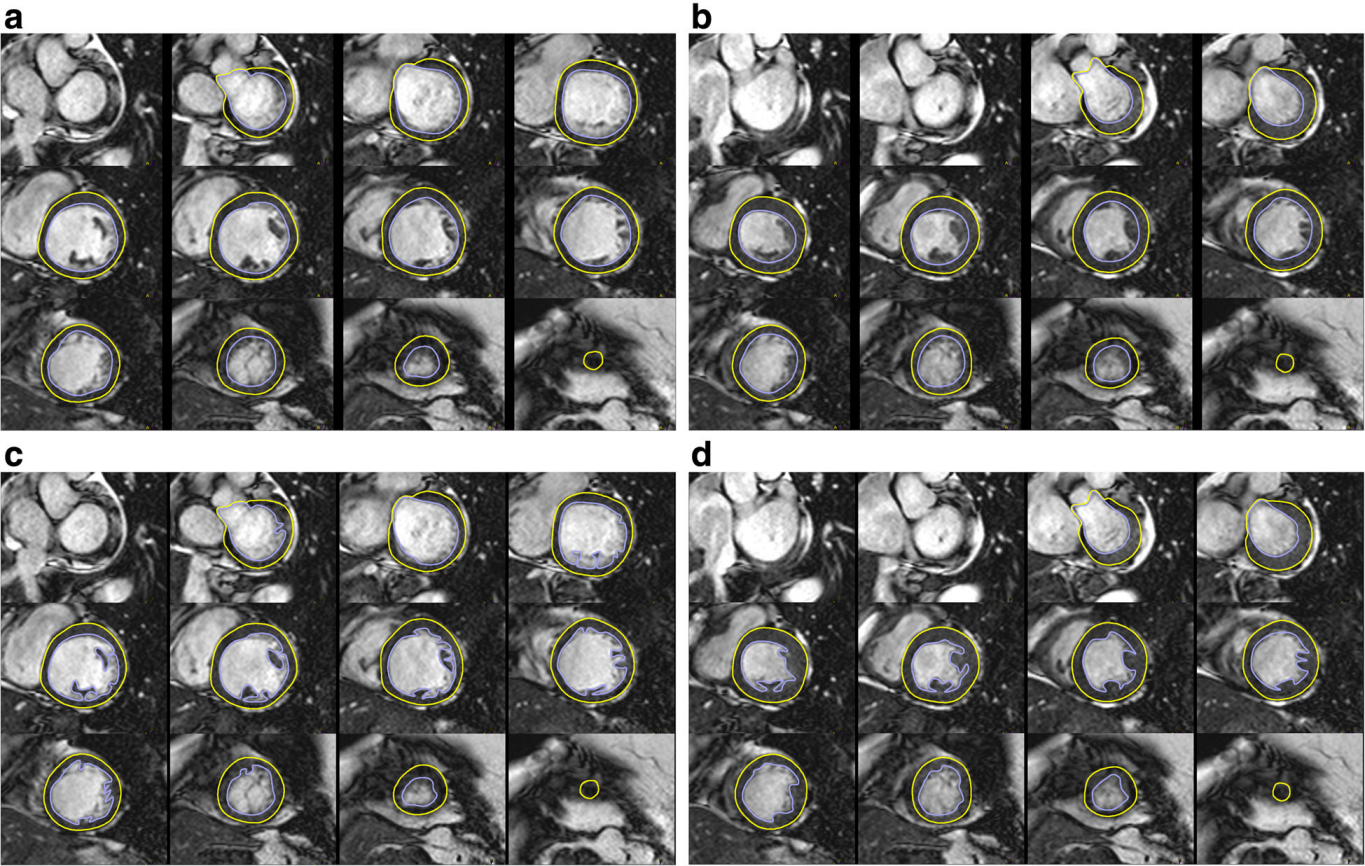
Left ventricular (LV) chamber quantification. For LV chamber quantification, the endocardial (blue) and epicardial (yellow) contours are delineated in diastole (left) and systole (right) in a stack of short axis slices that cover the whole left ventricle. **a** and **b** illustrate the approach with inclusion of the papillary muscles as part of the LV volume. **c** and **d** show the approach with exclusion of the papillary muscles from the LV volume

- General recommendations
  i) In patients with severe arrhythmias, the end-systolic volumes tend to be overestimated and ejection fraction underestimated. In case of significant artifacts this should be denoted in the report.
  ii) Calculated parameters: LV end-diastolic volume, LV end-systolic volume, LV stroke volume, LV ejection fraction, cardiac output, LV mass, and body-surface area indexed values of all except ejection fraction. The parameters quantified may vary depending on the clinical need.
  iii) Evaluation of the stack of short axis images with computer-aided analysis packages.
  iv) Contours of endocardial and epicardial borders at end-diastole and end-systole (Fig. 1).
  v) Epicardial borders should be drawn on the middle of the chemical shift artifact line (when present).
  vi) The LV end-diastolic image should be chosen as the image with the largest LV blood volume. For its identification, the full image stack should be be evaluated and one phase has to be identified as end-diastole for all the short axis locations. In addition, closure of the mitral valve or the phase immediately before opening of the aortic valve may be used for orientation.
  vii) The LV end-systolic image should be chosen as the image with the smallest LV blood volume. For its identification, the full image stack has to be evaluated and one phase has to be identified as end-systole for all the short axis locations.
  viii) Deviations may occur and extra care should be taken in the setting of LV dyssynchrony.
  ix) Automatic contour delineation algorithms must be checked for appropriateness by the reader.
- LV volumes
  i) Papillary muscles and trabecular tissue are myocardial tissue and thus ideally should be included with the myocardium as part of LV mass. As there is still discussion on the exact delineation of papillary muscles (e.g. versus trabeculation) and not all evaluation tools allow for their inclusion without manual drawing of contours, they are often included in the blood pool volume in clinical practice, which is acceptable. Reference ranges that use the same approach both on the acquisition and post-processing side must be used. (Fig. 1) [8–10].
  ii) Outflow tract: The LV outflow tract is included as part of the LV blood volume. When aortic valve cusps are identified on the basal slice(s) the contour is drawn to include the outflow tract to the level of the aortic valve cusps.
  iii) Basal descent: As a result of systolic motion of the mitral valve toward the apex (basal descent), care must be taken with the one or two most basal slices by using a standardized consistent approach. A slice that contains LV blood volume at end-diastole may include only left atrium (LA) without LV blood volume at end-systole. The LA can be identified by tracking wall thickening (if there is thickening - then it is in the LV cavity) and cavity (shrinking in systole, when in the cavity). Alternatively, the basal slice may be defined by at least 50% of the blood volume surrounded by myocardium. Currently however, there is no expert consensus on which method to use. Some software packages automatically adjust for systolic atrioventricular ring descent using cross-referencing from long axis locations.
- LV mass
  i) Calculation: difference between the total epicardial volume (sum of epicardial cross-sectional areas multiplied by the sum of the slice thickness and interslice gap) minus the total endocardial volume (sum of endocardial cross-sectional areas multiplied by the sum of the slice thickness and interslice gap), which is then multiplied by the specific density of myocardium (1.05 g/ml).
  ii) Papillary muscles: Papillary muscles and trabecular tissue are myocardial tissue and thus ideally should be included with the myocardium as part of LV mass, and this is particularly relevant in diseases with LV hypertrophy [6]. However, readers may decide to exclude trabecular tissue and papillary muscles from the myocardial mass. Reference ranges that use the same approach must be used (Fig. 1) [8–10].
  iii) Basal descent and apex: When the most basal slice contains only a small crescent of basal lateral myocardium and no discernable ventricular blood pool, an epicardial contour for the visible myocardium is included for LV mass only. Similarly, when the most apical slice contains only a circle of myocardium without cavitary blood pool, an epicardial contour without an endocardial contour should be drawn for LV mass calculations.
- Rapid quantitative analysis
  i) A rapid quantitative analysis, known as the area-length method, can be performed using biplanar (e.g. 2- and 4-chamber views) or rotational multiple long axis views. In cases without expected significant regional variation of wall motion, this technique allows for faster evaluation and is not limited by problems related to basal descent. However, the 4-chamber view is strongly influenced by breath-hold position. The accuracy is not similar to short axis coverage, but allows a fast analysis often more similar to transthoracic echocardiography results. When the area-length method is used, with either a single long-axis view or a biplane approach, specific mention of the analysis technique should be made in the report.
  ii) Calculation [11–13]:
    Single long-axis equation: LV volume = 0.85 × (LV-area)^2^/ LV-length. This is typically performed using a 4-chamber view with calculations of LV volume obtained on both end-diastolic and end-systolic phases. LV area is the planimetered area of the LV cavity from an endocardial contour with the base drawn as a straight line through the medial and lateral aspects of the mitral annulus. LV length is the linear dimension from the midpoint of the mitral annular line to the apical tip of the endocardial contour.
    Biplane equation: LV volume = 0.85 × (LV-area1 x LV-area2) / LV length. Here, both 4-chamber (LV-area1) and 2-chamber [or vertical] (LV-area2) long-axis views are used to calculate both end-diastolic and end-systolic volumes, similar to the single long-axis equation.
    Multi-plane long axis: A series of long-axis views rotating around the central longitudinal axis of the LV is used to calculate volumes. Six views provide results that do not differ from short-axis stacks [14].
- Cavity diameter and LV wall thickness can be obtained similar to echocardiography using two CMR approaches [12, 15]:
  i) Basal short-axis slice: immediately basal to the tips of the papillary muscles.
  ii) 3-chamber view: in the LV minor axis plane at the mitral chordae level basal to the tips of the papillary muscles.
  iii) Both approaches have good reproducibility. The 3-chamber view is most comparable to data obtained with echocardiography.
  iv) For maximal LV wall thickness, the measurement should be made perpendicular to the LV wall to ensure accurate measurements. At the apex, short-axis images are oblique to the axis of the wall and will be inaccurate. In this location in particular, long-axis views should be used.
- Research:
  i) Real-time cine acquisitions become increasingly available and might be beneficial in patients with arrhythmia or limited breathholding capacity. 3D cine acquisitions are also evolving to accelerate examination time. Post-processing of real-time images and 3D cine acquisitions is still technically evolving. The Task Force chooses to refrain from making a dedicated statement at this time.
  ii) Quantitative evaluation of LV myocardial dynamics (e.g., strain, rotation, time-to-peak velocity) is feasible by several imaging techniques (e.g., tagging, DENSE, SENC, tissue phase mapping, feature tracking) and requires specific post-processing software. As research applications are evolving and consensus evidence is being accumulated, the Task Force chooses to refrain from making a dedicated statement at this time.

## Right ventricular (RV) chamber assessment

### Visual analysis

- Review all cines in cine mode, validate observations from one plane with the others, and check for artifacts and coverage of the right ventricle (RV).
- Assessment of global and regional RV function (septal wall, free wall), where appropriate. Wall motion should be described as hyperkinetic, normokinetic, hypokinetic, akinetic, or dyskinetic. For qualitative regional analysis, wall motion in the RV free wall (e.g., basal, mid, and apical portions), outflow tract and inferior wall may be evaluated as relevant to the specific clinical scenario and diagnosis.
- Assessment of LV and RV chambers for hemodynamic interaction (i.e. constrictive physiology).

### Quantitative analysis

**Fig. 2:**
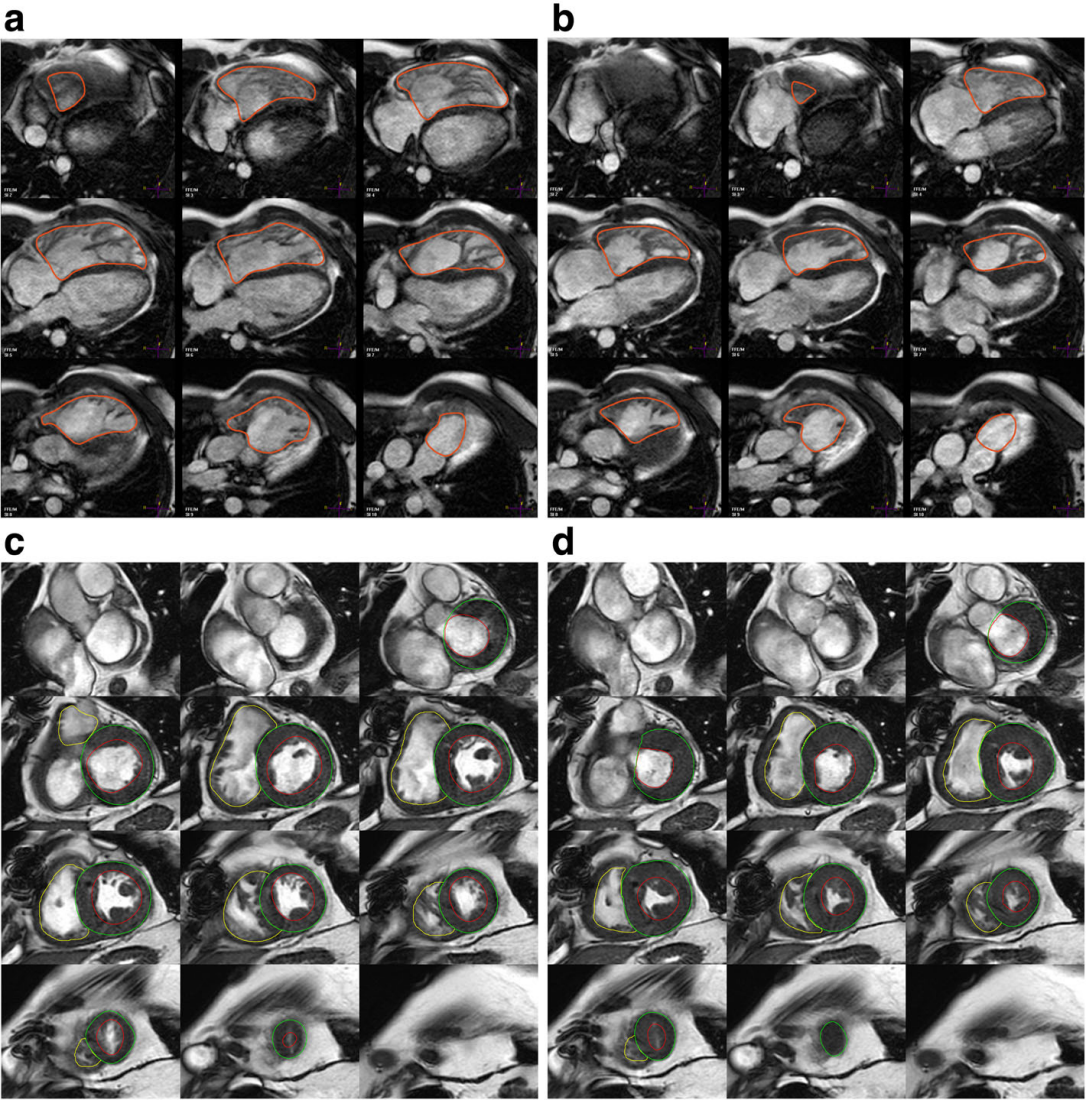
Right ventricular (RV) chamber quantification. For RV volume quantification, the endocardial (red) contours are delineated in diastole (**a**) and systole (**b**) in a stack of transaxial slices covering the whole RV (top). Alternatively, a stack of short axis slices can be used (**c**, **d**). Here, the yellow contours indicate the RV in diastole (**c**) and systole (**d**); the RV is contoured following the LV analysis (in **c** and **d**, red / green contours indicate endocardial / epicardial borders of the LV) and with reference to the LV

- General recommendations
  i) Calculated parameters: RV end-diastolic volume, RV end-systolic volume, RV ejection fraction, RV stroke volume, cardiac output, and body-surface area indexed values of all except ejection fraction. Similar to the LV, the parameters quantified may vary depending on the clinical need [16].
  ii) The contiguous stack of short-axis images or axial cine images is evaluated with computer-aided analysis packages (Fig. 2) [17, 18]. Automatically generated contours have to be carefully reviewed.
  iii) An axial stack of cines covering the RV provides the best identification of the tricuspid valve plane. A short-axis stack of cines is best for delineating the inferior wall.
  iv) Endocardial borders are contoured at end-diastole and end-systole (Fig. 2).
  v) The RV end-diastolic image should be chosen as the image with the largest RV blood volume. For its identification, the full image stack has to be evaluated and one phase has to be identified as end-diastole for all locations.
  vi) RV end-systolic image should be chosen as the image with the smallest RV blood volume. For its identification, the full image stack has to be evaluated and one phase has to be identified as end-systole for all slices.
  vii) As for the LV, it may be necessary to review all image slices in the stack to define end-systole.
  viii) The pulmonary valve may be visualized, and contours are included just up to, but not superior to this level.
- RV volumes
  i) Total volumes are taken as the sum of volumes from individual 2D slices, accounting for any interslice gap and slice thickness. RV trabeculae and papillary muscles are typically included in RV volumes.
- RV mass is usually not quantified in routine assessment. In selected patients, quantification of RV mass may be considered (e.g., in pulmonary hypertension).
- Confirmation of results
  i) If no shunts or valvular regurgitation is present, the RV and LV stroke volumes should be nearly equal (small differences are seen as a result of bronchial artery supply and papillary muscle inclusions in the measurements). Since the LV stroke volume is more reliably determined than the RV stroke volume, the LV data can be used to validate RV data.

## Post-processing of myocardial perfusion imaging

### Visual analysis

**Fig. 3:**
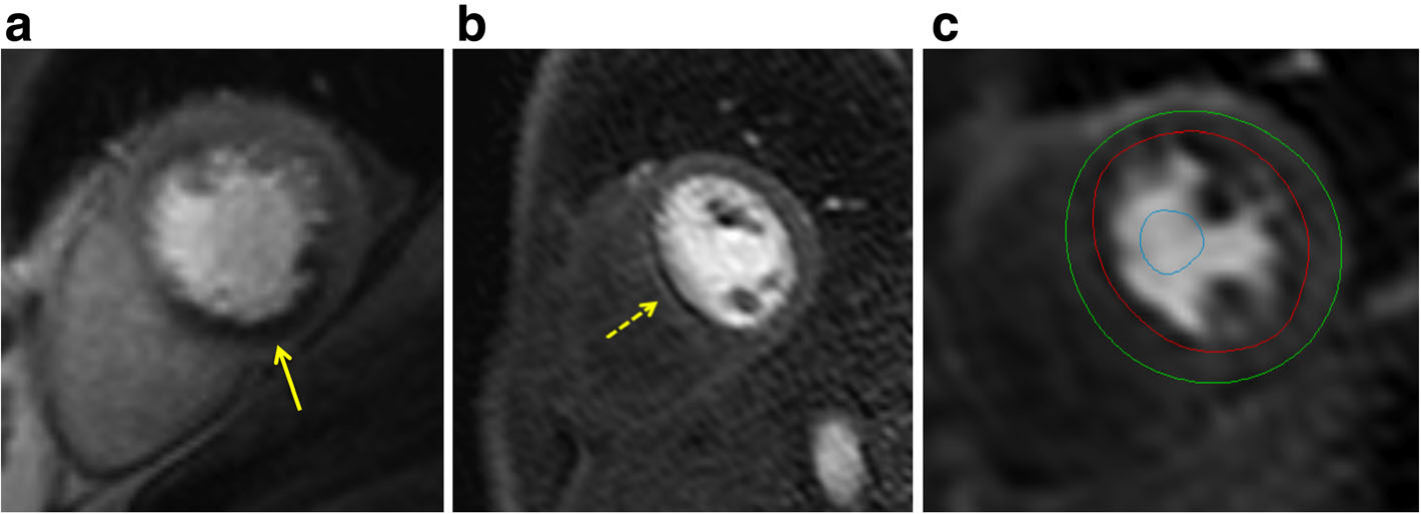
Perfusion imaging. **a** Perfusion defect in the inferior segments (yellow arrow). Note defect is predominantly subendocardial, affects the perfusion territory of the right coronary artery and is more than one pixel wide. **b** Dark banding artifact (yellow arrow). Note defect is very dark, occurs already before contrast reaches the myocardium, is seen in the phase encoding direction (right-left in this case), and is approximately one pixel wide. **c** Positioning of endocardial (red) and epicardial (green) contours and a region-of-interest (ROI) in the LV blood pool (blue) for semiquantitative or quantitative analysis of perfusion data

- Workflow:
  i) Display perfusion and corresponding LGE images side-by-side.
  ii) Adjust window, contrast and brightness level for an optimized contrast within the LV myocardium (not the entire image). The aim of image adjustment is to set a maximal window width without **“**spilling**”** of the LV cavity signal into the myocardium. Ensure that myocardium before contrast arrival is nearly black and that the window settings maximize the contrast within the myocardium. Note that that the correct level and window settings requires review of both pre- and peak contrast images.
  iii) Apply the same contrast, brightness and window settings to all images of the dynamic series.
  iv) Review series as cines and/or by scrolling through individual images.
  v) Check that there was an adequate haemodynamic response to stress by reviewing the heart rate and blood pressure change between stress and symptomatic response to stress. Images may also be checked for ‘splenic switch off’ during stress [19].
  vi) The key diagnostic feature for identifying a perfusion defect is the arrival and first passage of the contrast bolus through the LV myocardium.
  vii) Visual analysis is based on a comparison between regions to identify relative hypoperfusion. Comparison should be made between endocardial and epicardial regions, between segments of the same slice and between slices.
- Stress images alone may permit the diagnosis of inducible perfusion defects. When the diagnosis is unclear based on stress images alone and rest images are available, these two image series can be compared. In general, an inducible perfusion defect will be present on the stress, but not the rest images. If perfusion defects are seen on both stress and rest images, they may be artifacts or have other causes such as myocardial scar. Note that artifacts may be less pronounced or absent on rest compared with stress images due to differences in haemodynamics and contrast kinetics between stress and rest.
- Scar tissue may not necessarily cause a perfusion defect, especially if rest perfusion is acquired after stress. Scar should therefore always be identified from LGE and not from perfusion images.
- Criteria for an inducible perfusion defect (Fig. 3a):
  i) Occurs first when contrast arrives in LV myocardium.
  ii) Persists beyond peak myocardial enhancement and for several RR intervals.
  iii) Is more than two pixels wide.
  iv) Is usually most prominent in the subendocardial portion of the myocardium.
  v) Often manifests as a transmural gradient across the wall thickness of the segment involved: most dense in the endocardium and gradually becoming less dense towards the epicardium.
  vi) Over time, defect regresses from the subepicardium towards the subendocardium.
  vii) Is present at stress but not at rest.
  viii) Conforms to the distribution territory of one or more coronary arteries.
- Interpret location and extent of inducible perfusion defect(s) using AHA segment model [5].
  i) Comment on transmurality of perfusion defect [20].
  ii) Indicate extent of perfusion defect relative to scar on LGE.
- Criteria for dark banding artifacts (Fig. 3b): A common source of false-positive reports are subendocardial dark banding artifacts [21]. These artifacts have the following characteristics:

- Are most prominent when contrast arrives in the LV blood pool.
- Lead to a reduction in signal compared with baseline myocardial signal whereas a true perfusion defect does not show a decrease in signal compared with baseline. These subtle differences can be hard to appreciate visually. It can therefore be helpful to draw a region of interest (ROI) around the suspected artifact and display its SI-time profile.
- Persist only transiently before the peak myocardial contrast enhancement.
- Appear predominantly in the phase-encoding direction.
- Are approximately one pixel wide.
  Dark banding present at stress and at rest with no corresponding scar on LGE images is also indicative of an artifact [22]. Note however that differences in heart rate and baseline contrast can change the appearance and presence of dark banding between stress and rest perfusion images. Thus, absence of dark banding at rest with typical dark banding at stress should not on its own be considered diagnostic for an inducible perfusion defect.

g) Pitfalls of visual analysis
  i) Multi-vessel disease: Visual analysis is based on relative signal differences within an imaged section of the heart. Theoretically, the presence of balanced multivessel disease can result in most or all of the imaged section appearing hypoperfused, which can lead to false-negative readings and needs to be considered in relevant clinical circumstances. In practice, however, truly balanced ischaemia is rare and a perfusion defect in one or more territories will be more prominent. Even if all coronary territories are affected, the severity of the observed defects typically is more pronounced around the geographic center(s) of the coronary territories. In addition, a clear endocardial to epicardial signal gradient is usually seen in multi-vessel disease [23]. Quantitative analysis of the dynamic perfusion data may be of further help to detect globally reduced myocardial perfusion reserve in multi-vessel disease.
  ii) Microvascular disease: Diseases that affect the myocardial microvasculature (e.g., diabetes mellitus, systemic hypertension) may lead to a global subendocardial reduction in perfusion [24–27]. This can lead to false-positive readings relative to angiographic methods and needs to be considered in relevant clinical circumstances. Features suggesting microvascular disease are the presence of concentric LV hypertrophy and a concentric, often subendocardial perfusion defect crossing coronary territories. Differentiation from multi-vessel disease can be challenging.
  iii) If vasodilation during stress data acquisition was inadequate, visual analysis may lead to false negative interpretation of the perfusion study [28].
  iv) The distance of the myocardium to the surface coil affects signal intensity and may lead to misinterpretation if not considered in the analysis. These problems are less likely if acquisition is corrected for coil sensitivity.

### Quantitative analysis

- A quantitative analysis of the SI change in myocardial perfusion CMR studies can be performed. Several methods have been described for this purpose. In clinical practice, these are rarely required, but they may supplement visual analysis for example in suspected multi-vessel disease or suspected inadequate response to vasodilator stress. Fully automated methods for quantitative perfusion analysis are becoming available and may soon become more widely used. Quantitative analysis is also frequently used in research studies.
- Requirements:
  i) Validation and definition of a normal range with the specific pulse sequence and contrast regime used for data acquisition. If only a comparison between regions of the same study is made, establishing a normal range is less relevant.
  ii) A temporal resolution of one RR interval is recommended.
  iii) Consideration of potential saturation effects (higher contrast agent doses are more likely to lead to saturation effects).
- Semi-quantitative analysis:
  i) Analysis methods that describe characteristics of the SI profile of myocardial perfusion CMR studies without estimating myocardial blood flow are typically referred to as “semi-quantitative analysis methods”.
  ii) Workflow:
    Select an image from the dynamic series with good contrast between all cardiac compartments (some post-processing tools generate an average image of the series).
    Outline LV endocardial and epicardial contours on this image (manual or automated) (Fig. 3c).
    Propagate contours to all other dynamic images.
    Correct contour position for in-plane motion (some analysis packages register images prior to contours being outlined).
    Depending on the type of analysis to be performed, place a separate ROI in the LV blood pool. Preferably, the basal slice is used. Exclude papillary muscles and flow artifacts from the ROI.
    Select a reference point in the LV myocardium for segmentation (usually one of the RV insertion points) [5].
    Segment LV myocardium according to AHA classification [5]
    Generate SI / time profiles for myocardial segments +/− LV blood pool.
    Consider generating division into endocardial and epicardial layers and repeat analysis [20].
  iii) Frequently used semi-quantitative analysis methods (see [29] for detailed review):
    Maximal upslope of the myocardial SI profile, may be normalized to LV upslope [30].
    Time to peak SI of the myocardial SI profile [31, 32].
    Ratio of stress/rest values for the above (often referred to as “myocardial perfusion reserve index”) [33, 34].
    The upslope integral (area under the signal intensity-time curve) [35].
  iv) Limitations of semi-quantitative analysis methods:
    SI may vary according to distance from coil. This can be partially corrected by using a pre-contrast proton density image or other coil sensitivity correction tools.
    No absolute measurement of myocardial blood flow given.
- Quantitative analysis
  i) Analysis methods that process the SI profile of myocardial perfusion CMR studies to derive estimates of myocardial blood flow are typically referred to as “quantitative analysis methods” [29, 36, 37].
  ii) Requirements:
    It is a prerequisite for reliable quantification that data acquisition used an appropriate pulse sequence and contrast regime.
    The requirements for the acquisition method depend on the analysis method. Currently, this typically requires at least a proton density image, the generation of an input function which is not saturated by using dual bolus [38] or dual contrast [39].
    Motion correction to correct for respiratory motion is preferable.
  iii) Workflow:
    Manual analysis methods require contour placement as described above for semi-quantitative analysis. Dynamic SI data are then typically exported to off-line workstations for further processing.
    Fully automated methods are becoming available, which generate pixel-wise maps of myocardial perfusion without user input.
  iv) Several analysis methods have been described, including:
    Model-based methods [40, 41].
    Model-independent methods [42, 43].

## Post-processing of late gadolinium enhancement (LGE) of the left ventricle

### Visual assessment

**Fig. 4:**
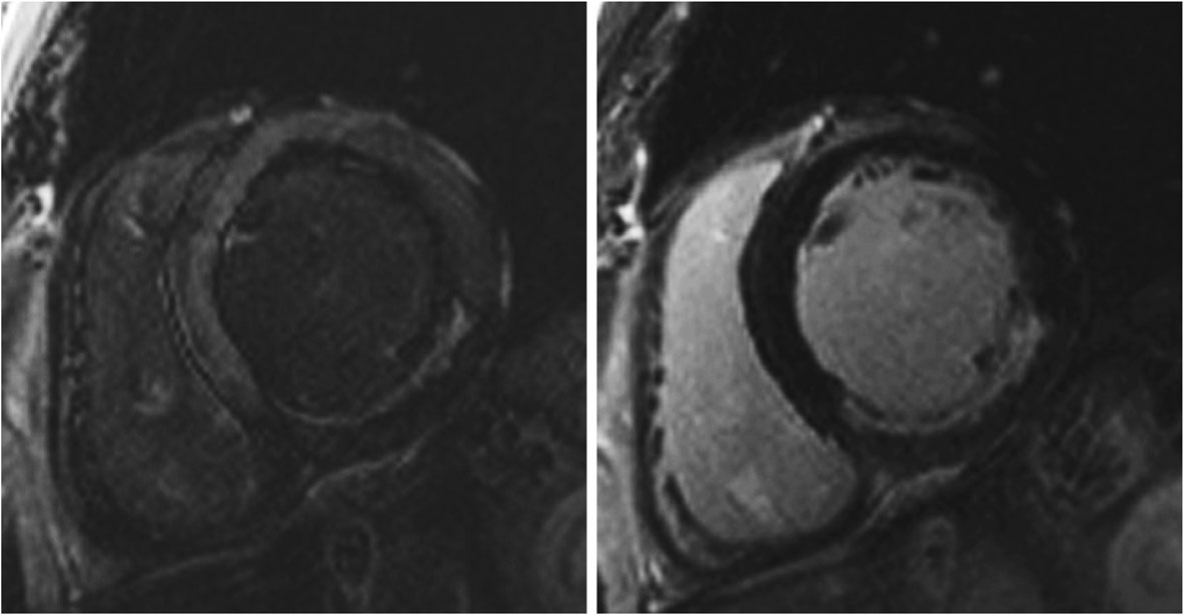
Late gadolinium enhancement (LGE) imaging. Role of inversion time in LGE imaging: On the left panel which is a magnitude (non-PSIR) LGE image, normal myocardium has a faint **“**etched**”** appearance (darkest at the border with higher signal intensity centrally) signifying an inversion time that was set too short and which will lead to underestimation of LGE. On the right panel, the image was repeated with a longer inversion time and demonstrates a larger LGE zone in the inferior wall. For non-PSIR magnitude imaging, always use the longest inversion time possible that still nulls normal myocardium

- For most clinical indications, visual assessment of LGE images is sufficient.
- Workflow:
  i) Modify image window and level so that:
    Noise is still detectable (nulled myocardium should not be a single image intensity).
    LGE regions are not saturated (LGE regions should not be a single image intensity).
  ii) Note, on magnitude (not phase-sensitive inversion recovery [PSIR]) images, if normal myocardium has a faint “etched” appearance (darkest at the border with slightly higher image intensity centrally), this signifies an inversion time that was set too short and will lead to underestimation of the true extent of LGE (Fig. 4). In general, an inversion time that is slightly too long is preferred to one that is slightly too short [44].
- Criteria for presence of LGE.
  i) High SI area that is visibly brighter than “nulled” myocardium.
  ii) Verify regions with LGE in at least one other orthogonal plane and/or in the same plane obtain a second image after changing the direction of readout.
- Assess pattern of LGE
  i) Coronary artery disease (CAD) type: Should involve the subendocardium and be consistent with a coronary artery perfusion territory.
  ii) Non-CAD-type: Usually spares the subendocardium and is limited to the mid-wall or epicardium, although non-CAD-type should be considered if subendocardial involvement is global [45].
- Interpret location and extent using AHA 17-segment model [5] [20].
  i) Comparison of LGE images should be made with cine and perfusion images (if the latter are obtained) to correctly categorize ischemia and viability [46].
  ii) Estimate average transmural extent of LGE within each segment (0%, 1–25%, 26–50%, 51–75%, 76–100%) [44].
  iii) In patients with acute myocardial infarction, include subendocardial and mid-myocardial hypoenhanced no-reflow zones as part of infarct size.
- Pitfalls
  i) Bright ghosting artifacts can result from poor electrocardiogram (ECG) gating, poor breath-holding, and long T1 species in the imaging plane (e.g., cerebrospinal fluid, pleural effusion, gastric fluid, etc.) [47]
  ii) On non-PSIR images, tissue with long T1 (regions below the zero-crossing) may appear enhanced [44, 48].
  iii) Occasionally, it can be difficult to distinguish no reflow zones or mural thrombus from viable myocardium. Imaging using a long-inversion time [49], using PSIR, or performing post-contrast cine imaging may be helpful in this regard.
  iv) In case of reduced contrast, the interpretation of additional sequences may be necessary (see below section “Dark-blood/grey blood LGE”).
  v) In PSIR images manual windowing and quantification algorithms may behave differently when compared with magnitude images.

### Quantitative analysis

- Quantitative analysis is primarily performed to measure LGE extent and/or grey-zone extent for research purposes. Subjective visual assessment is still a prerequisite to identify poor nulling, artifacts, no-reflow zones, etc., and to draw endocardial and epicardial borders.
- Multiple different methods of delineating LGE extent are described in the literature including the following: manual planimetry, the n-SD technique, and the full width half maximum (FWHM) technique. As the research applications are evolving and consensus evidence is being accumulated, the Task Force chooses to refrain from making a dedicated statement at this time regarding the *optimal* method for quantitative assessment [50–55].

### Research tools / quantitative analysis

- Quantification of LGE extent:
  i) Manual planimetry:
    Outline endocardial and epicardial borders.
    Manual planimetry of LGE regions in each slice.
    Summation of LGE areas.
    Multiplication of total LGE area with slice thickness plus interslice gap as well as specific gravity of myocardium provides the approximate LGE mass, which can be used to calculate the ratio of LGE to total myocardial mass.
    Considered subjective.
    Adjustment for regions with intermediate signal intensities (grey zones) caused by partial volume can improve reproducibility of measurements [54].
  ii) The n-SD technique:
    Manual outlining of endocardial and epicardial borders for the myocardial ROI.
    Manual selection of a normal “remote” (dark) region ROI within the myocardium to define the reference SI (mean and standard deviation, SD). This subjective approach can affect measurements.
    It is susceptible to spatial variations in surface coil sensitivity.
    Selection of a threshold between normal myocardium and LGE. The relative SNR of scar tissue versus normal myocardium can vary dependent on contrast agent type, dose and time after injection, field strength, type of sequence and other variables including the underlying injury itself. As such, there is no cutoff value which works for all situations and usually manual tracing is performed as the standard of truth. But (semi-) automated thresholding may improve reproducibility after adequate standardization. As a starting point for semiautomatic thresholding we recommend 5-SD for infarction. There is currently not enough evidence to provide a cut-off for non-ischemic LGE.
    The presence of LGE within the myocardium is then determined automatically.
    Requires manual corrections to include no-reflow zones and to exclude artifacts and LV blood pool (errors in the endocardial contour).
  iii) FWHM technique:
    Manual outlining of endocardial and epicardial borders for the myocardial ROI.
    Uses the full width of the myocardial ROI SI histogram at half the maximal signal within the scar as the threshold between normal myocardium and LGE.
    Visual determination whether LGE is present or not, and, if LGE is present, manual selection of a ROI that includes the region of “maximum” signal. This subjective selection can affect measurements.
    Is also susceptible to spatial variations in surface coil sensitivity, albeit perhaps less so than the n-SD technique [51].
    Considered more reproducible than the n-SD technique [53].
    Since the technique assumes a bright LGE core, it may be less accurate than the n-SD technique if LGE is patchy or grey [56].
    Requires manual corrections to include no-reflow zones and to exclude artifacts and LV blood pool (errors in the endocardial contour).
- Peri-infarct zone:
  Multiple methods for quantifying the extent of the peri-infarct or grey zones are reported [57, 58].
  The Task Force does not endorse any specific evaluation technique due to the strong impact of partial volume effects.
- Dark-blood/grey blood LGE
  Multiple techniques are described in the literature but one that is “flow-independent”, (i.e., does not rely on blood flow to suppress blood-pool signal) is preferable [59–61].
  As the research application(s) are evolving and consensus evidence is being accumulated, the writing group chooses to refrain from making a dedicated statement at this time regarding the optimal method for quantitative assessment of dark-blood/grey blood LGE images.
- LGE in other chambers than LV

There is increasing evidence about LGE imaging of the RV, which is usually captured with standard LGE protocols imaging the LV. Imaging the thin LA wall is difficult and requires specialized sequences. As the applications are evolving and consensus evidence is being accumulated, the writing group chooses to refrain from making a dedicated statement at this time regarding the post-processing assessment of LGE in chambers other than the LV.

## Post-processing of T1 mapping

### Background

**Fig. 5:**
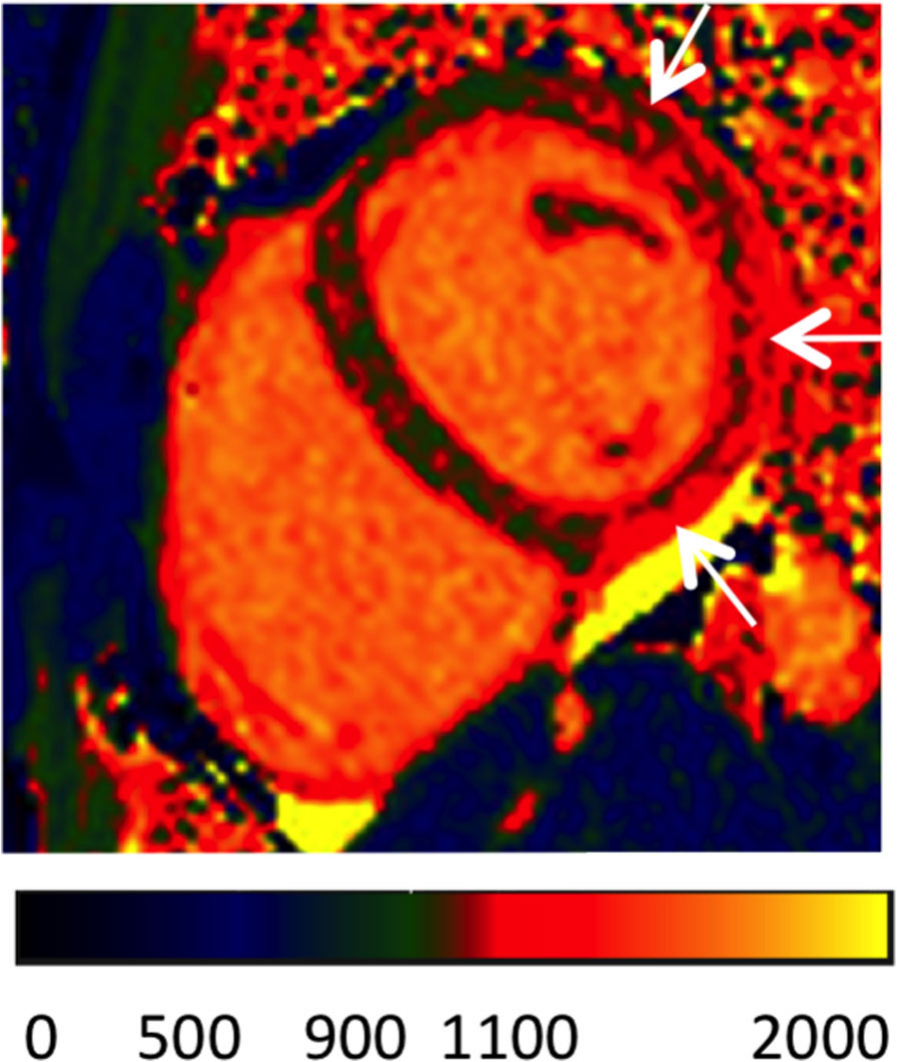
Native T1 map in a patient with acute myocarditis illustrating T1 elevation in the subepicardial lateral LV wall (modified from [64])

In 2013, the “T1 Mapping Development Group” published a consensus statement that proposed suitable terminology and specific recommendations for site preparation, scan types, scan planning and acquisition, quality control, visualization and analysis, and technical directions [62]. Building on this initiative, the Consensus Group on Cardiac MR Mapping has formed itself and published in 2017 “Clinical recommendations for CMR mapping of T1, T2, T2* and extracellular volume: A consensus statement by the Society for Cardiovascular Magnetic Resonance (SCMR) endorsed by the European Association for Cardiovascular Imaging (EACVI)” [63]. The following recommendations refer to these consensus statements. For more details regarding when and how to use T1 mapping, refer to this original consensus statement as well as to the SCMR protocol recommendations (Fig. 5).

### Visual analysis

- The visual analysis of the series of differently T1-weighted source images should aim to detect and verify diagnostic image quality.
- The visual analysis of the final T1 map should aim to detect artifacts and verify diagnostic image quality. Automatically generated quality control maps (e.g., T1*) may be used to exclude misregistration or significant artifacts.
- Maps may be displayed in color if the pertinent look-up tables are set according to site-specific ranges of normal, or in gray scale in combination with appropriate image processing, to highlight areas of abnormality.

### Quantitative analysis

- For global assessment and diffuse disease, a single ROI should be drawn conservatively in the septum on mid-cavity short-axis maps to reduce the impact of susceptibility artifacts from adjacent tissues.
- In case of artifacts or inconclusive results obtained from mid-cavity ROIs, basal ROIs can be used for validation.
- For focal disease, additional ROIs might be drawn in areas of abnormal appearance on visual inspection. A very small ROI (< 20 pixels) should be avoided.
- The position and size of automatically generated ROIs should be validated.
- Drawing ROIs on greyscale images rather than color maps may reduce bias.
- For assessing diffuse disease, focal fibrosis as assessed by LGE imaging should be excluded from the ROI.
- There is currently no specific recommended / preferred analysis software package. The image reader should be trained with the local standards and with the analysis software package of choice and be familiar with the appearance of artifacts.
- The sensitivity of mapping techniques to confounders such as heart rate and magnetic field inhomogeneities should be considered during interpretation.
- Extracellular volume (ECV) estimation requires T1 mapping acquisitions before contrast agent administration (native T1) and after contrast agent administration (typically > 10 min post-contrast to approach steady-state conditions). The proposed post-processing steps should be applied equally to both maps.
- For calculating ECV, a ROI in the center of the blood pool in the native and in the post-contrast T1 map should be drawn excluding papillary muscles and trabeculae.
- For calculating ECV, hematocrit of the same day should be available. If this is not available, hematocrit may be estimated from native values of blood pool T1 (“synthetic ECV”) [65].
- ECV is given in %. The formula for calculating ECV:

$$ ECV=\frac{\left(\frac{1}{T1 my{o}_{postGd}}\frac{1}{T1 my{o}_{native}}\right)}{\left(\frac{1}{T1 bloo{d}_{postGd}}\frac{1}{T1 bloo{d}_{native}}\right)}\times \left(100- hematocrit\right) $$

m) Inline ECV maps can be a useful alternative to manual ECV calculations. The raw images should be checked to verify a diagnostic image quality and processing.
n) For clinical reports, the type of pulse sequence, reference range, and type/dose of gadolinium contrast agent (if applied) should be quoted.
o) Mapping results should include the numerical absolute value, the Z-score (number of standard deviations by which the result differs from the local normal mean; if available), and the normal range of the CMR system.
p) Local results should be benchmarked against published reported ranges, but a local reference range should be primarily used.
q) Reference ranges should be generated from data sets that were acquired, processed, and analyzed in the same way as the intended application, with the upper and lower range of normal defined by the mean ± 2 SD of the normal data, respectively.
r) Parameter values should only be compared to other parameter values if they are obtained under similar conditions. In other words, the acquisition scheme, field strength, contrast agent and processing approach should be the same, and the results should be reported along with corresponding reference ranges for the given methodology.

## Post-processing of T2-weighted imaging

### Visual analysis

**Fig. 6:**
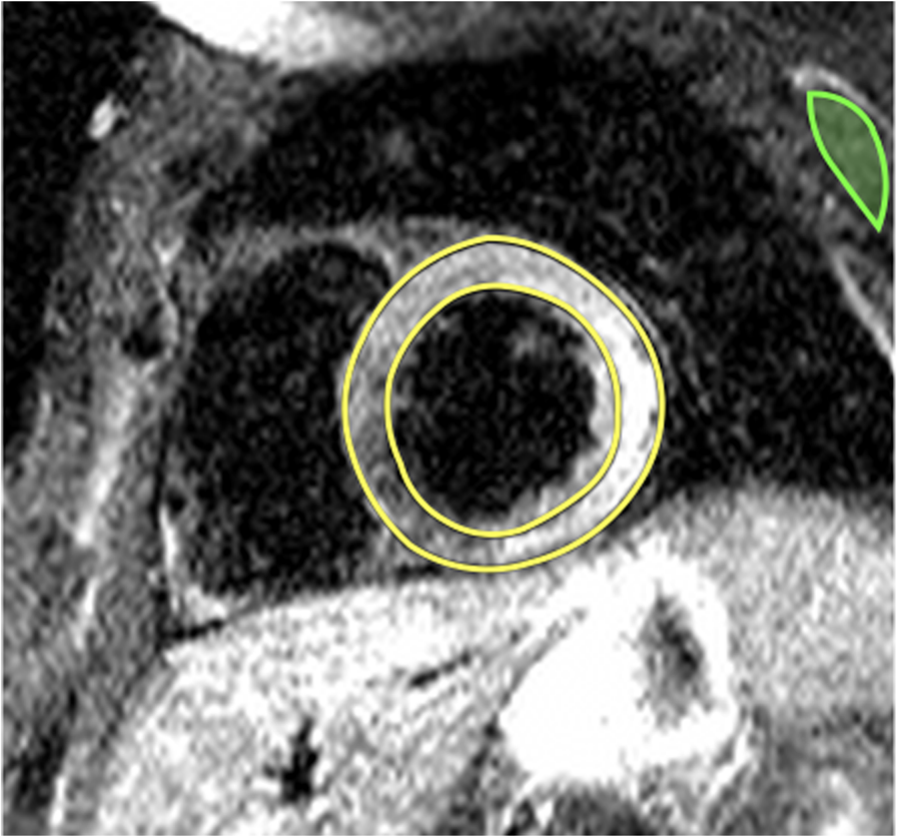
T2-weighted image (short-tau inversion recovery, STIR) in a midventricular short axis view with increased SI in the inferolateral and lateral segments in acute myocarditis

- The visual analysis of T2-weighted images aims for detecting or excluding regions with significant SI increase, as a marker for an increased free water content (edema).
- Qualitative, visual analysis of myocardial SI may be sufficient for diseases with significant regional injury to the myocardium, such as acute ischemic injury/infarction, acute myocarditis (Fig. 6), stress-induced (Takotsubo) cardiomyopathy, and sarcoidosis.
- Workflow:
  i) Identify and display appropriate image(s).
  ii) Modify image contrast and brightness in the myocardial tissue to minimize SI in the most normal appearing myocardium (noise should still be detectable there) and to maximize the maximal SI displayed in the myocardium area without allowing for **“**over-shining”, i.e., displaying non-white pixels as white.
  iii) Check for artifacts (typically SI changes crossing anatomical structures).
- Criteria for edema:
  i) Clearly detectable high SI area respecting anatomical borders.
  ii) Follows an expected regional distribution pattern (transmural, subendocardial, subepicardial, focal).
  iii) Verifiable in two perpendicular views.
- High SI areas suggestive of myocardial edema should be compared to
  i) regional function.
  ii) other tissue pathology such as scar/fibrosis and infiltration.
- Pitfalls of visual analysis:
  i) Surface coil reception field inhomogeneity: The uneven distribution of the sensitivity of the receiving surface coil may lead to falsely low SI in segments distant to the coil or falsely high SI in segments close to the coil surface, especially in dark-blood triple-inversion recovery spin echo (STIR, TIRM) images. If no efficient SI correction algorithm for balancing the signal intensity across the reception field is available, the body coil, albeit with a lower signal-to-noise ratio, provides a more homogeneous signal reception.
  ii) Low SI artifacts: Arrhythmia or through-plane motion of myocardium may cause artifacts, making areas appear with falsely low SI, especially in dark-blood triple-inversion recovery spin echo images.
  iii) High SI artifacts: In dark-blood triple-inversion recovery spin echo images, slow flowing blood may lead to insufficient flow suppression and results in high SI of blood, typically along the subendocardial border. This can be confused with myocardial edema.

### Semi-quantitative analysis

- Because low SI artifacts can lead to SI distribution patterns that may mimic extensive myocardial edema, a mere visual analysis may lead to incorrect results. SI quantification with reference regions is much less sensitive to these errors and therefore is recommended.
- Requirements:
  i) Tested normal values for SI values or ratios.
- Workflow
  i) Global SI analysis:
    Outline LV endocardial and epicardial contours.
    For the T2 SI ratio, draw the contour for a ROI in a large area of the skeletal muscle closest to the heart and to the center of the reception field of the coil (for short axis views preferably in the *M. serratus* anterior [66].
  ii) Regional SI analysis:
    Draw the contour for a ROI in the affected area and divide the SI by that of the skeletal muscle.
  iii) While a cut-off of 1.9 can be used for dark blood triple-inversion recovery spin echo [67], a locally established value is recommended, because SI and ratio values may vary between sequence settings (especially echo time (TE)) and CMR scanner models. For these images, a color-coded map, based on the parametric calculation and display of myocardial pixels with a SI ratio of 2 or higher, can also be used.

## Post-processing of T2 mapping

### Background

The Consensus Group on Cardiac MR Mapping published in 2017 “Clinical recommendations for CMR mapping of T1, T2, T2* and extracellular volume: A consensus statement by the Society for Cardiovascular Magnetic Resonance (SCMR) endorsed by the European Association for Cardiovascular Imaging (EACVI)” [63]. The following recommendations refer to this consensus statement. For more details regarding when and how to use T2 mapping, refer to this original consensus statement as well as to the SCMR protocol recommendations.

### Visual analysis

- The visual analysis of the series of differently T2-weighted source images should aim for detecting and excluding artifacts and significant motion.
- The visual analysis of the final T2 map should aim for detecting and excluding artifacts.
- Maps may be displayed in color if the color look up tables are set according to site-specific ranges of normal, or in gray scale in combination with appropriate image processing, to highlight areas of abnormality.

### Quantitative analysis

- For global assessment and diffuse disease, a single ROI should be drawn conservatively in the septum on mid-cavity short-axis maps to reduce the impact of susceptibility artifacts from adjacent tissues.
- In case of artifacts or non-conclusive results on mid-cavity ROIs, basal ROIs can be used for validation.
- For focal disease, additional ROIs might be drawn in areas of abnormal appearance on visual inspection. Very small ROIs (< 20 pixels) should be avoided.
- ROIs should be checked if generated automatically.
- Drawing ROIs on greyscale instead of color maps may avoid bias.
- Depending on the goal of the analysis, focal fibrosis as assessed by LGE imaging may be excluded from the ROI.
- There is currently no specific preferred analysis software package. The image reader should be trained with the local standards and with the analysis software package of choice and be aware of and familiar with the appearance of artifacts.
- Sensitivity of mapping techniques to confounders such as heart rate and magnetic field inhomogeneities should be considered during interpretation.
- Mapping results should include the numerical absolute value, the Z-score (number of standard deviations by which the result differs from the local normal mean), and the normal reference range.
- Parameter values should only be compared to other parameter values if they are obtained under similar conditions. In other words, the acquisition scheme, field strength and processing approach should be the same, and the results should be reported along with corresponding reference ranges for the given methodology.

## Post-processing of T2* imaging

### Visual analysis

T2* imaging always requires a quantitative analysis. Visual analysis is used to ensure adequate image quality, which is the most important factor for the accuracy of data analysis.

### Quantitative analysis

**Fig. 7:**
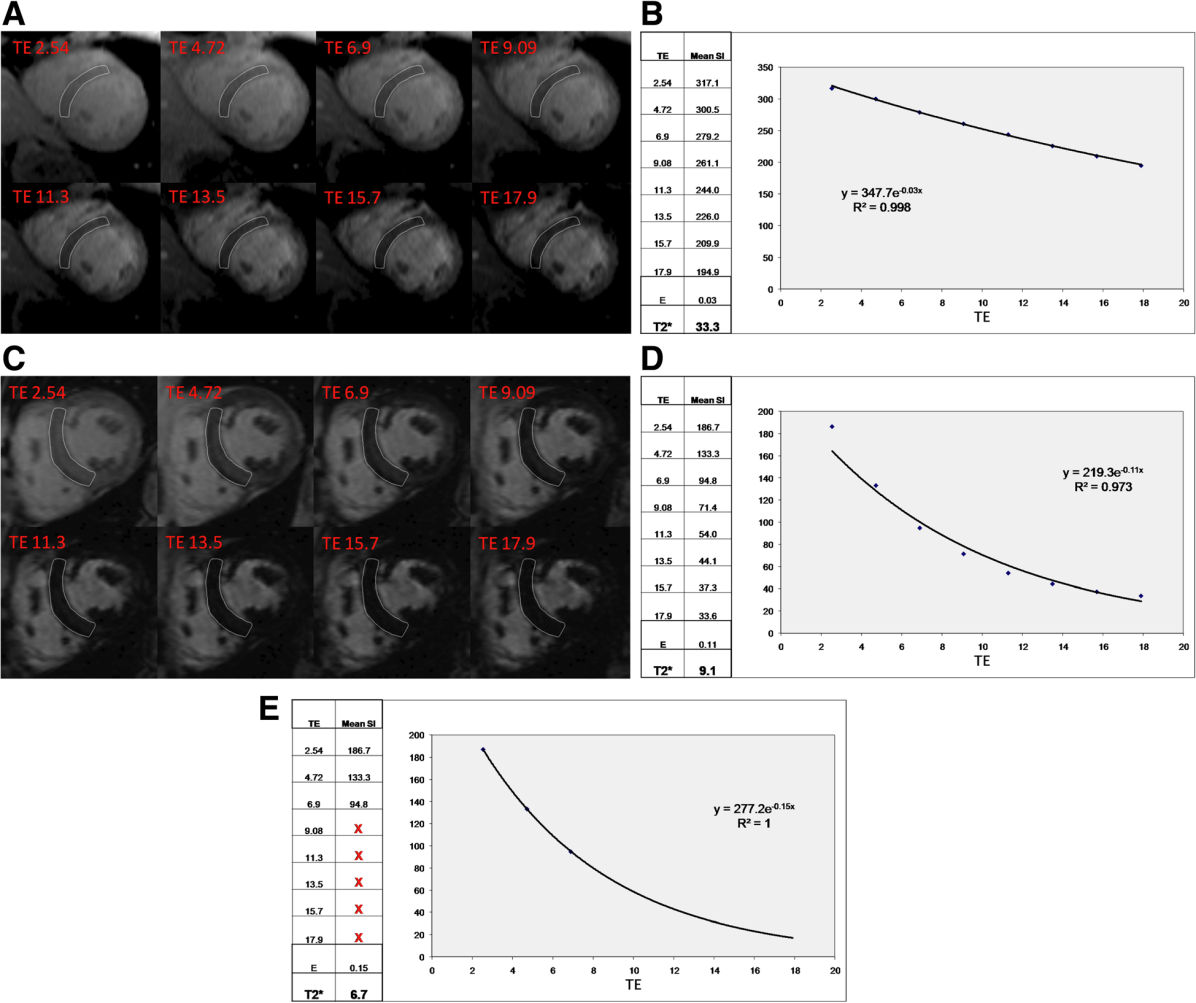
T2* imaging to assess myocardial iron overload. **a** T2* scan of a normal heart showing slow signal loss with increasing TE. **b** Decay curve for normal heart. T2* = 33.3 ms. **c** Heavily iron overloaded heart. Note there is substantial signal loss at TE = 9.09. **d** Decay curve for heavily iron overloaded heart showing rapid signal loss with increasing TE. The curve plateaus as myocardial SI falls below background noise. **e** Values for higher TEs are removed (truncation method) resulting in a better curve fit and a lower T2* value

- Evaluation of T2* always requires a quantitative analysis using software with regulatory approval for T2* evaluation in patients.
- Full thickness ROI located in the ventricular septum
  i) Septal ROI is drawn on mid-LV short-axis image.
  ii) Take care to avoid blood pool and proximal blood vessels.
  iii) A septal ROI avoids susceptibility artifact from tissue interfaces.
- Mean myocardial SI from the ROI is plotted against TE (Fig. 7)
  i) SI falls with increasing TE.
  ii) Curve fitting should apply a validated algorithm.
  iii) The time for the decay of SI falls (shorter T2*) with increasing iron burden.
  iv) In heavily iron overloaded patients, SI for higher TEs may fall below background noise causing the curve to plateau and underestimating T2*.
  v) This can be compensated for by:
    Truncating the curve by removing later echo times (Fig. 7e) [68, 69].
    This issue is not significant when using the double inversion recovery (black blood) sequence [70].
- Cut-off values at 1.5 Tesla:
  i) Normal cardiac T2* is 40 ms [71]
  ii) T2* < 20 ms indicates cardiac iron overload [72]
  iii) T2* < 10 ms indicates increased risk of development of heart failure [73]
- CMR assessment of T2* at 3 T for assessment of iron overload cardiomyopathy cannot be recommended at this time. T2* shortens with increasing field strength making assessment of severe iron overload more problematic, and there is a lack of clinical verification.

## Flow image interpretation and post-processing

### Background

CMR flow imaging provides information about blood flow velocities and volumes, and enables the visualization of blood flow. Flow assessment in a 2D slice is in widespread use. Recently, temporally resolved flow evaluation in a 3D volume (4D flow) has evolved enormously. It is currently predominantly used for evaluating congenital heart disease. For further details regarding application, acquisition and postprocessing of 4D flow also refer to the corresponding consensus document [74].

### Visual analysis

- Appropriately aligned acquisitions of cines and stacks of cines can give valuable information on flow in relation to adjacent structures, notably on the directions, time courses and approximate dimensions of jets resulting from valve regurgitation, stenoses or shunts. Such information can be important in assessing the credibility of measurements of flow, which may be subject to several possible sources of error. Gradient echo cines differ somewhat from balanced steady state free precession (bSSFP) in terms of degrees of signal augmentation or reduction attributable to flow effects. Of note, bSSFP can provide clear delineation between the relatively bright signal from voxels aligned within the coherent core of a jet, and low signal from the shear layer that bounds such a jet core. In- or through-plane phase contrast flow velocity acquisitions can also provide visual information on the directions, dimensions and time courses of flow; it can also image morphology, which can yield a clue to the etiology of an abnormal jet [75, 76]. It is also often used in congenital heart disease. Color flow mapping in post-processing software may be useful in determining directionality of the jet or morphology.
- Pitfalls:
  i) Flow appearances on both cine and phase encoded acquisitions are highly dependent on image location and orientation, especially in the case of jet flow.
  ii) Check for the appropriate velocity encoding. If the range of velocity encoding (VENC) is set too high, visualization of the jet may not be obtained and may be inaccurate as well as having poorer SNR. If it is set too low, a mosaic pattern on the images will be visualized [77].
  iii) If slice thickness is too large on in- or through-plane velocity mapping, the higher velocities will be “averaged out**”** with the lower velocities and stationary tissue; jets and flow may not be visualized correctly.
  iv) If the annulus of valves is very dynamic or the imaging plane is not set correctly, the valve morphology may not be visualized.
  v) If imaging in the presence of metal containing devices, signal loss may be present as artifact and interpretation must proceed with caution.
  vi) Check for appropriate spatial and temporal resolution. For spatial resolution, 8 to 16 pixels should fill the vessel to obtain accurate results on through-plane velocity mapping. For temporal resolution, there should be at least 11–16 frames per cardiac cycle [78].

### Quantitative analysis

**Fig. 8:**
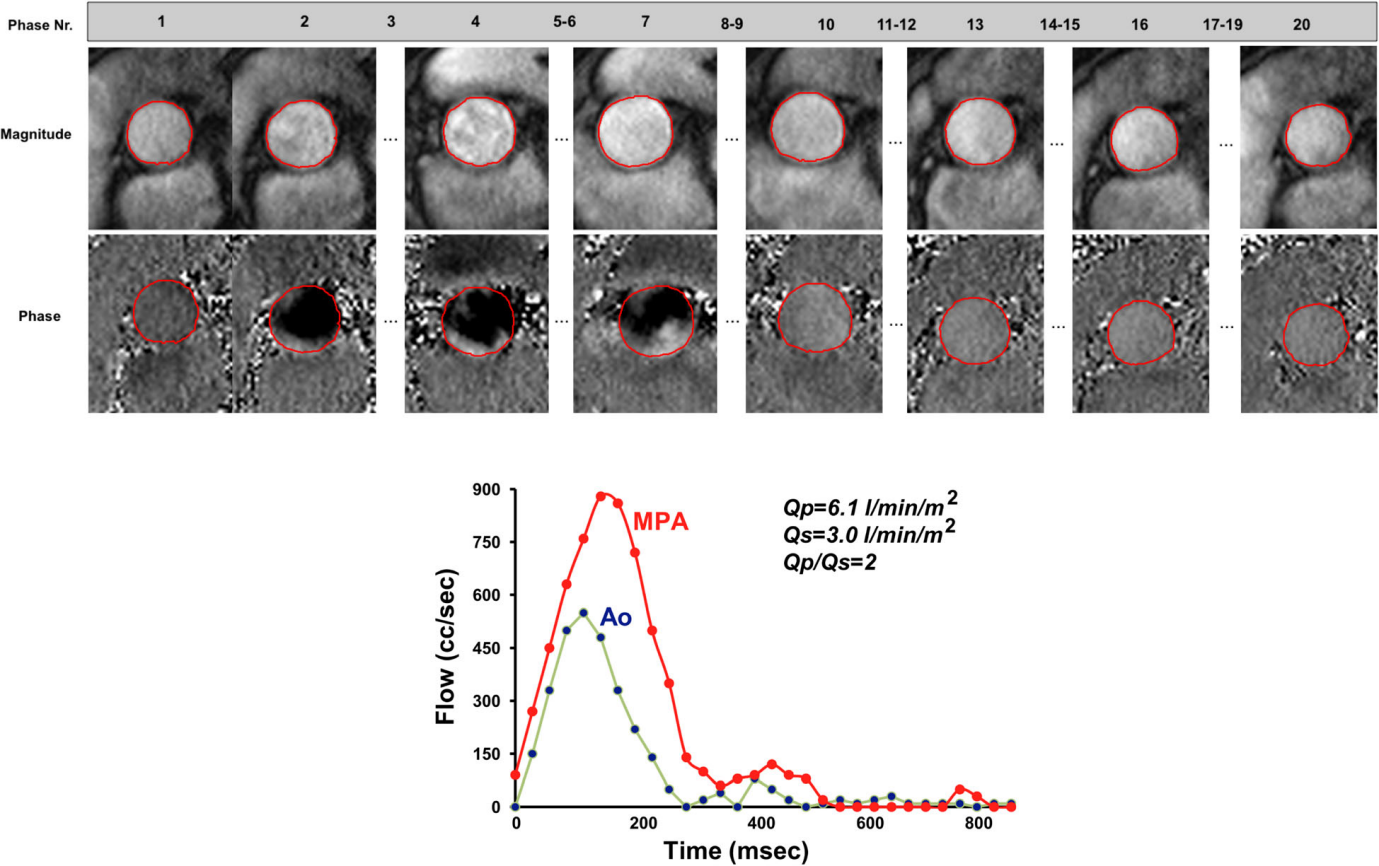
Quantification of blood flow. (top) Contours were drawn delineating the aortic lumen at the sinotubular level during all 20 phases of the cardiac cycle to assess aortic flow. (bottom) Flow curves from measurements in the ascending aorta and in the pulmonary artery in a patient with ventricular septal defect showing a left-to-right shunt

- Workflow:
  i) Through-plane measurements may be supplemented by in-plane measures if needed.
  ii) Review phase and magnitude images side by side. Window the magnitude and phase images to the appropriate brightness and contrast so that the borders of the ROI are sharp.
  iii) Examine the images to ensure the quality is sufficient and that the VENC was not exceeded, or there is little contrast (i.e., the VENC was too high).
  iv) Trace the borders of the vessel of interest on each phase and magnitude image so that only the cavity of the vessel is included (Fig. 8); make sure the noise outside the vessel is not included. Check that this is performed correctly on the magnitude images always keeping in mind that it is the phase images that contain the encoded information.
  v) Baseline-correction may be considered. As the utility and exact methods are not yet established, the writing group chooses to refrain from making a dedicated statement at this time regarding its use.
  vi) Directly calculated parameters include antegrade and retrograde volume, flow rate, peak and mean velocity.
  vii) Derived parameters include:
    Net volume [ml| = antegrade volume - retrograde volume
    Regurgitant fraction [%] = (retrograde volume / antegrade volume) * 100.
    Cardiac output (liters/min = (net volume [ml] x heart rate [beats/minute])/1000) and cardiac index (cardiac output/body surface area) when integrating heart rate and body surface area (BSA)
    Regional flow to both lungs by measuring cardiac output in each branch pulmonary artery (e.g., percentage of flow to the right lung = (right pulmonary artery flow / right pulmonary artery flow + left pulmonary artery flow) Å~ 100).
    Regurgitant volumes of the atrioventricular valves may be obtained by either of 2 methods: A) direct measurement of diastolic flow across the valve and subtraction of systolic forward flow across the associated semilunar valve or B) measurement of ventricular stroke volume using cine CMR and subtraction of forward flow across the associated semilunar valve.
    Quantitative aortic regurgitant volume may be inaccurate in the presence of a large, dilated aorta. An alternative is to subtract net pulmonary artery flow or the sum of caval return from the forward flow across the aortic valve in the absence of significant aortic to pulmonary collateral flow (noting that this will be a slight overestimate as bronchial flow is ~ 5% if total aortic output) [79].
    Estimation of cardiac shunts is feasible by calculating Qp/Qs based on the stroke volume obtained by flow measurements in the pulmonary artery and at the aortic sinutubular junction. Shunts can also be quantified by direct measurement of the flow through the shunt.
- Pitfalls:
  i) On the phase images, the area of flow may be slightly larger than the area of the magnitude images. Care has to be taken when evaluating the magnitude images – the size of the ROI has to be adapted.
  ii) If the VENC is exceeded, some software packages allow for adjusting the “dynamic range” of the velocity scale so that the VENC is not exceeded. For example, if the peak velocity in the aorta is 175 cm/s and the VENC was set at 150 cm/s, the dynamic range is between − 150 cm/s and + 150 cm/s (i.e., 300 cm/s). This may be moved to − 100 cm/s and + 200 cm/s to account for this accelerated velocity. This will be demonstrated on the graph of the velocity where the phase in which the VENC is exceeded does not “alias” (appears to go the wrong way) after correction.
  iii) In general, the area that exceeds the VENC in the ROI is in the center of the vessel and not at the edges; if it is at the edges, it is usually (but not always) outside the vessel.
  iv) If imaging in the presence of devices, signal loss may be present as artifact and interpretation must proceed with caution [80].
  v) When measuring peak velocity, some software packages will determine the peak velocity in one pixel in the ROI whereas others may take the peak velocity of the average of a few adjacent pixels in the ROI. By reporting the peak velocity in a single pixel, noise may make this measurement inaccurate. By reporting this as an average of a few adjacent pixels, noise is less of an issue, however, the true peak velocity may be higher than the reported value. These factors must be kept in mind and interpretation may need to be adapted to the measurement technique used.
  vi) When attempting to measure peak velocity using through-plane velocity mapping along a vessel, interpretation should be tempered by the notion that this parameter may be an underestimate as the true peak velocity lies somewhere along the vena contracta; the through-plane velocity map may not have been obtained at the level of the true peak velocity. If the vena contracta is itself narrow or ill defined, jet velocity mapping is unlikely to be possible.
  vii) Peak velocity is only minimally affected by small background phase offsets, while volume measurements can be dramatically affected by even a small background phase offset due to the cumulative aspect of integration overspace (within the ROI) and time (over the cardiac cycle). Dilatation of a vessel tends to increase error of this type [81].
  viii) Orientation of the image plane perpendicular to flow direction can have a significant impact on peak velocity measurement, while not significantly affecting volume flow [78].
  ix) Internal consistency may be used to partially assess the accuracy of measurement (e.g., the sum of the flows in the branch pulmonary arteries should sum to the flow in the main pulmonary artery, and comparing the stroke volume obtained by flow measurement with the stroke volume obtained by volumetry of cine images).

### Research tools

- Real time velocity mapping: The utility and post-processing algorithm best applied to this approach is the subject of ongoing research.

## Post-processing of angiography of thoracic aorta, pulmonary arteries and veins

### Visual analysis

**Fig. 9:**
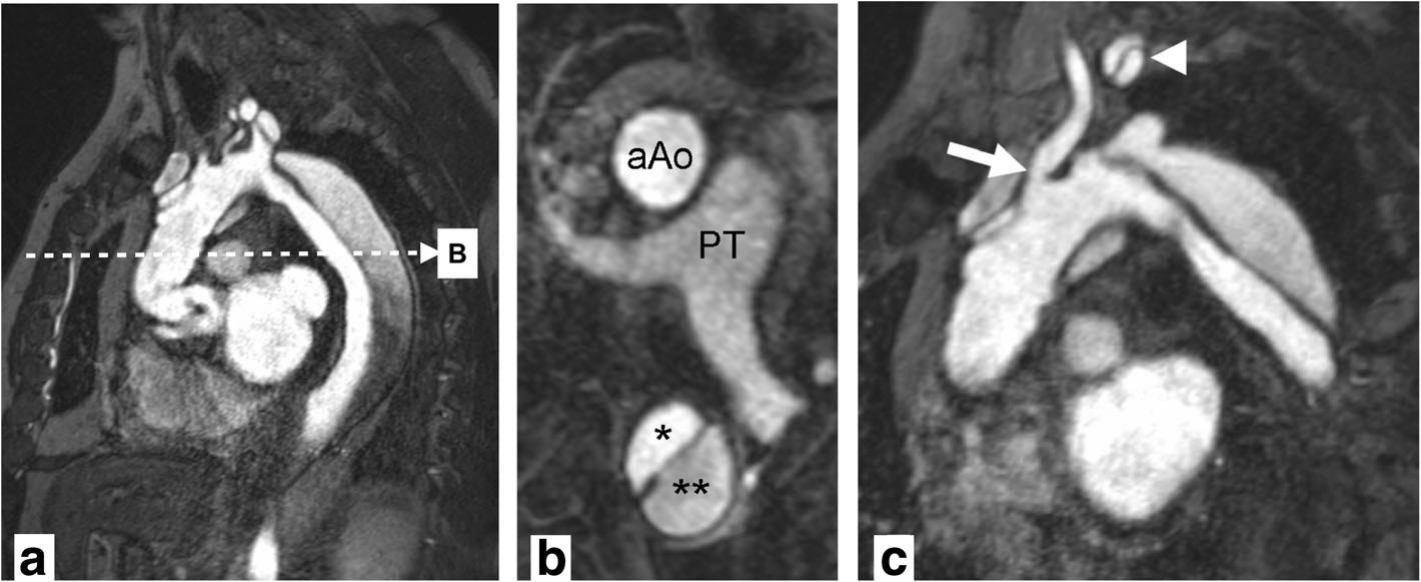
Magnetic resonance angiography. Stanford A aortic dissection after surgical repair with graft of ascending aorta. Panel **a** shows a source image of breath-held 3D gradient recalled echo sequence after contrast injection. Multiplanar reformats in axial orientation (**b**) at the level of the pulmonary trunk (PT) show a normally perfused ascending aorta graft (aAo) and persistent dissection in descending aorta with true (*) and false (**) lumina. Double oblique reformat (**c**) shows narrowing at the origin of the left common carotid artery (arrow) and dissection membrane propagating into the left subclavian artery (arrowhead) with perfusion of both lumina

- MIP for first review of 3D data and for demonstration purposes (Fig. 9). Volume rendered (VR) techniques may be used for demonstration purposes, but not for quantitative analysis.
- Aorta [82, 83]:
  i) Wall thickness: Review bSSFP or turbo spin echo images. Avoid measurement in areas with artifacts that may distort anatomy, such as chemical shift artifacts.
  ii) Wall irregularities: Review 3D-MRA source images and bSSFP or turbo spin echo.
- Pulmonary arteries [84]:
  i) Multiplanar double oblique and targeted MIP reconstructions for assessment of wall adherent thrombi, emboli, wall irregularities and abrupt diameter changes.
- Pulmonary veins [85]:
  i) Assess for atypical insertion, small accessory veins and ostial stenoses.
- Coronary arteries:
  i) Coronary MRA (either contrast-enhanced or non-contrast MRA using 3D whole heart bSSFP) can play a role in assessment of congenital anomalies [86], but not usually in the context of ischemic heart disease. The origins, branching patterns, and course of coronary arteries is best assessed on source images, MPR or targeted MIP reconstructions.

### Quantitative analysis

**Fig. 10:**
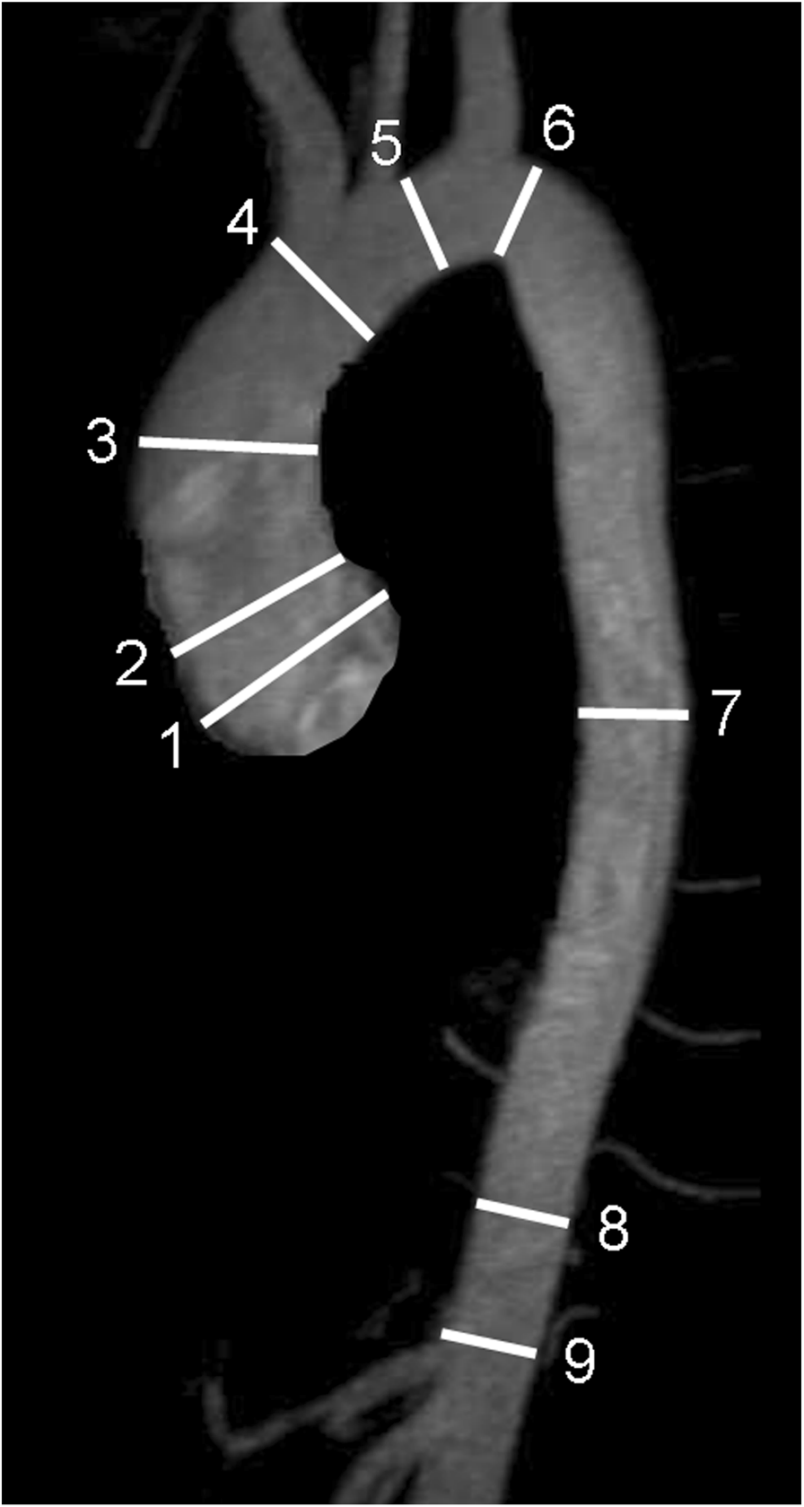
Anatomic landmarks for standardized reporting of diameters of the aorta at the level of sinuses of Valsalva (1), sinotubular junction (2), mid-ascending aorta (3), proximal to brachiocephalic trunk (4), between left common carotid and left subclavian arteries (5), distal to left subclavian artery (6), mid-descending aorta (7), diaphragm (8), abdominal aorta above celiac trunk (9). (Adapted from [87])

- Aorta:
  i) Diameters of the aorta are measured on double oblique MPR of source images perpendicular to the vessel centerline at standardized levels (Fig. 10) [87]. In oval shaped vessels the longest diameter and its perpendicular diameter shall be reported. Both, inner (lumen) or outer (external vessel wall) diameter may be measured. This should be included in the report, as well as the type of angiography (with or without contrast-enhancement). Measurement of outer contour is recommended in dilation such as in aneurysms, while the inner contour is recommended in the setting of stenosis, such as in coarctation.
  ii) In the presence of wall thickening (e.g. thrombus or intramural hematoma) inner and outer diameter including vessel wall thickness should be reported.
  iii) Aortic root measurements require ECG-gated images. Diameter of the sinus portion should be recorded as the maximum sinus to sinus measurement perpendicular to the vessel centerline. For more details and normal values refer to [82].
  iv) Standardized structured reports with tables of diameters are helpful for reporting follow-up examinations.
- Pulmonary artery:
  i) Diameters are measured on double oblique images perpendicular to the centerlines of the pulmonary trunk as well as right and left pulmonary arteries. It should be reported whether the inner or outer contour was measured. In oval shaped vessels the longest diameter and its perpendicular diameter shall be reported, with measurement during systole recommended. Alternatively, cross-sectional area may be measured. For normal values refer to [84].
- Pulmonary veins:
  i) Double oblique MPR of pulmonary veins perpendicular to centerline for diameter measurements. For a more comprehensive assessment including flow measurements refer to [85].

## Data Availability

not applicable

## Abbreviations

ACCF: American College of Cardiology Foundation
ACR: American College of Radiology
AHA: American Heart Association
ASE: American Society of Echocardiography
ASNC: American Society of Nuclear Cardiology
BSA: Body surface area
bSSFP: Balanced steady state free precession
CAD: Coronary artery disease
CMR: Cardiovascular magnetic resonance
DICOM: Digital imaging and communications in medicine
EACVI: European Association of Cardiovascular Imaging
ECG: Electrocardiogram
ECV: Extracellular volume
FWHM: Full width half maximum
HRS: Heart Rhythm Society
LA: Left atrium/left atrial
LGE: Late Gadolinium enhancement
LV: Left ventricle
MIP: Maximum intensity projection
MPR: Multiplanar reconstruction
MRA: Magnetic resonance angiography
NASCI: North American Society for Coronary Angiography and Intervention
PACS: Picture archiving and communications system
PSIR: Phase sensitive inversion recovery
ROI: Region of interest
RSNA: Radiological Society of North America
RV: Right ventricle/right ventricular
SCAI: Society of Cardiac Angiography and Intervention
SCMR: Society for Cardiovascular Magnetic Resonance
SD: Standard deviation
SI: Signal intensity
STIR: Short-tau inversion recovery
TE: Echo time
TI: Inversion time
TR: Repetition time
TSE: Turbo spin echo
VENC: Velocity encoding
VR: Volume rendering
